# Development of Promising Flower-like Ag/SrFeO_3_ Nanosheet Electrode Materials: An Efficient and Selective Electrocatalytic Detection of Caffeic Acid in Coffee and Green Tea

**DOI:** 10.1021/acsomega.3c03060

**Published:** 2023-11-22

**Authors:** Girija Kesavan, Thirumalairajan Subramaniam, Hariharan Vaiyapuri Manemaran

**Affiliations:** † Department of Physics, Dr. N.G.P. Arts and Science College, Coimbatore 641 048, India; ‡ Centre for Agricultural Nanotechnology, Tamilnadu Agricultural University, Coimbatore 641003, India

## Abstract

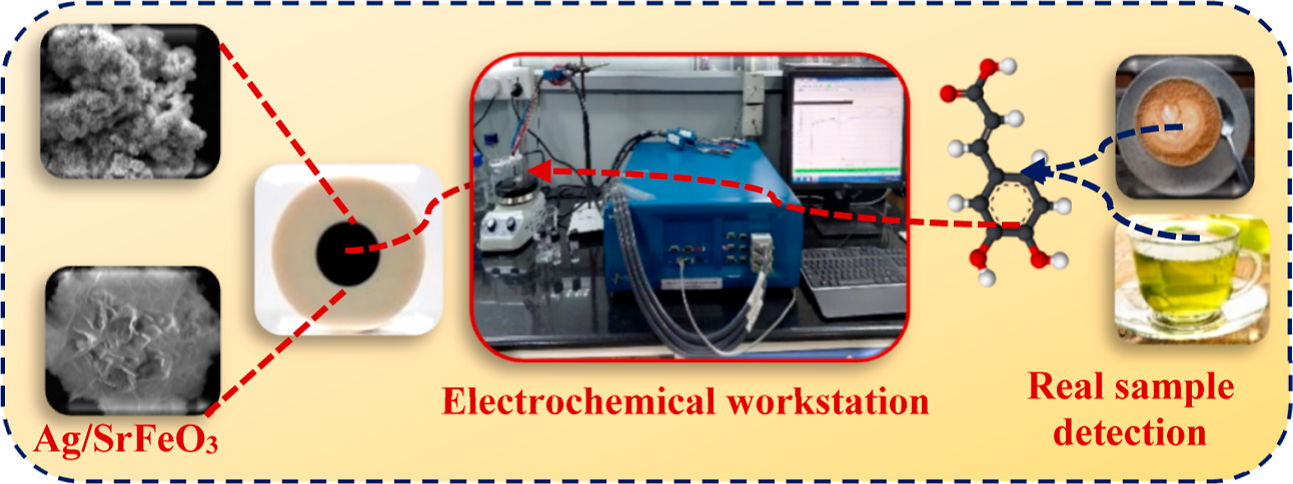

The development of highly efficient electrocatalytic sensors is necessary for detection in various paramedical and industrial applications. Motivated by this concept, we demonstrate flower-like Ag/SrFeO_3_ nanostructures prepared by a facile route to modify electrocatalyst material for the detection of caffeic acid (CA). The surface morphology, phase structure, particle size, and pore volume were investigated through different physicochemical analytical techniques. The cyclic voltammetry technique was employed to evaluate the electrochemical behavior of both glassy carbon and modified Ag/SrFeO_3_ electrodes toward CA. The study revealed that the modified electrode shows excellent electrocatalytic activity toward CA compared to the reported values, with a wide linear range of 1–15 nM, a detection limit of 23 nM, good stability, and excellent repeatability. The superior results are attributed to numerous factors such as rapid electron transfer ability, tunable texture, high surface area, and good conductivity. The created Ag/SrFeO_3_ nanostructure-based electrochemical biosensor is a potential candidate for real-time analytical performance to directly detect CA in commercially available coffee and green tea without any pre-treatment.

## 1 Introduction

In recent times, phenolic compounds have significant attention in the chemical, nutrient, and biological fields owing to their outstanding properties.^1^ Caffeic acid (CA) is one of the crucial phenolic compounds found in various fruits, vegetables, and hot and soft drinks, including broccoli, citrus fruits, tea, coffee, wines, and so forth.^2^ The molecular structure of CA contains two hydroxyl groups, which contribute significantly to its unique antioxidant properties, numerous pharmaceutical activities, and an extremely important role in human life, such as antiallergic, antibacterial, and antitumor effects.^3,4^ Production of CA is common in all plant species as a metabolite of hydroxycinnamate and phenylpropanoid. It is usually present in the everyday human diet and in a variety of beverages, including brewed coffee at 0.13 mg per 100 mL, 0.18 mg per 100 mL in green tea, 2 mg per 100 mL in red wine, 8 mg per 100 g in sunflower, about 5 mg per 100 mL in apple sauce, apricots, and prunes, and so forth.^5−7^ The CA ingestion level is required to maintain the human diet, and the suitable dosage listed by the National Food Consumption Survey (NFCS) is 0.3–0.5 g per day.^8^ However, excessive intake of CA may cause side effects and negative impacts on human health, such as the prevention and treatment of diseases related to inflammatory reactions, viral infections, oxidative stress, brain tissue damage, and immune regulation diseases.^8−10^ Therefore, there is a crucial and urgent need for low-level detection of CA in food-related samples.

To date, several instrumental analytical techniques have been used in the determination of CA, such as mass spectrometry, HPLC, capillary electrophoresis, and so forth.^11^ Notably, Cai et al. designed a fluorometric assay platform for the fluorescence detection of CA.^12^ Khezeli et al. detected the CA by a green ultrasonic-assisted liquid–liquid microextraction based on deep eutectic solvents.^13^ CA was measured by supercritical fluid, as reported by Konar et al.^14^ These aforementioned techniques had challenges, which included high costs, expensive instrumental setups, and a more time-consuming process. The development of analytical devices that combine high sensitivity, low-cost instrumentation, and quick detection is still a challenge. In order to overcome such problems, the electrochemical process has been recognized as a promising detection technique due to its high selectivity, fast response, on-site inspection, miniaturization, and ultrasensitive detection even at low concentrations of the target analyte. Nanostructured materials such as metal oxide, mixed metal oxide, perovskite oxide, and carbon-based materials can exhibit the desired properties for electrochemical biosensor applications.^15^ Surprisingly, surface plasmonic materials (Ag, Au, Cu, etc.) with perovskite nanostructure compositions mostly exhibit high conductivity, outstanding chemical stabilities, numerous active sites, and specific oxidation and reduction processes.^16−18^ Moreover, the composition within the high surface area and fast charge transport between the Ag and perovskite oxide supply plenty of pathways for electrochemical reactions. The main disadvantage of outmoded materials is low selectivity and overlapping, stability, and repeatability.^19^ For this reason, electrocatalyst materials are developed to enrich the wide-ranging performance of novel electrodes. A continuous effort is still being made to search for better electrocatalyst detection of CA to satisfy global needs.

In this present work, to the best of our knowledge, there are no reports available on the design of flower-like Ag/SrFeO_3_ nanostructure electrodes in electrocatalytic applications for the detection of CA. The prepared nanostructures were scientifically confirmed by their physicochemical properties using microscopic and spectroscopic techniques. The modified Ag/SrFeO_3_ electrode improves the electrocatalyst sensor for high sensitivity, a wide linear range, and good selectivity owing to enriching the conductivity and electron transfer rate, providing functional active sites, and effective surface area. Finally, the construction of a new electrochemical sensor was successfully applied to test CA in real samples of coffee and green tea with good recovery results without any pre-treatment.

## 2 Experimental Section

### 2.1 Chemicals and Reagents

All chemicals and reagents were purchased from Sigma-Aldrich, like silver nitrate (AgNO_3_), strontium nitrate [Sr(NO_3_)], sodium dodecyl sulfate (NaC_12_H_25_SO_4_), and CA. The supporting electrolyte was phosphate buffer solution (PBS), which was made with 0.1 M Na_2_HPO_4_·6H_2_O and NaH_2_PO_4_. NaOH was used to alter the pH of the electrolyte. The analyte was created using purified double-distilled water. All electrochemical studies were performed at ambient temperature.

### 2.2 Preparation of Ag/SrFeO_3_

**Figure 1:**
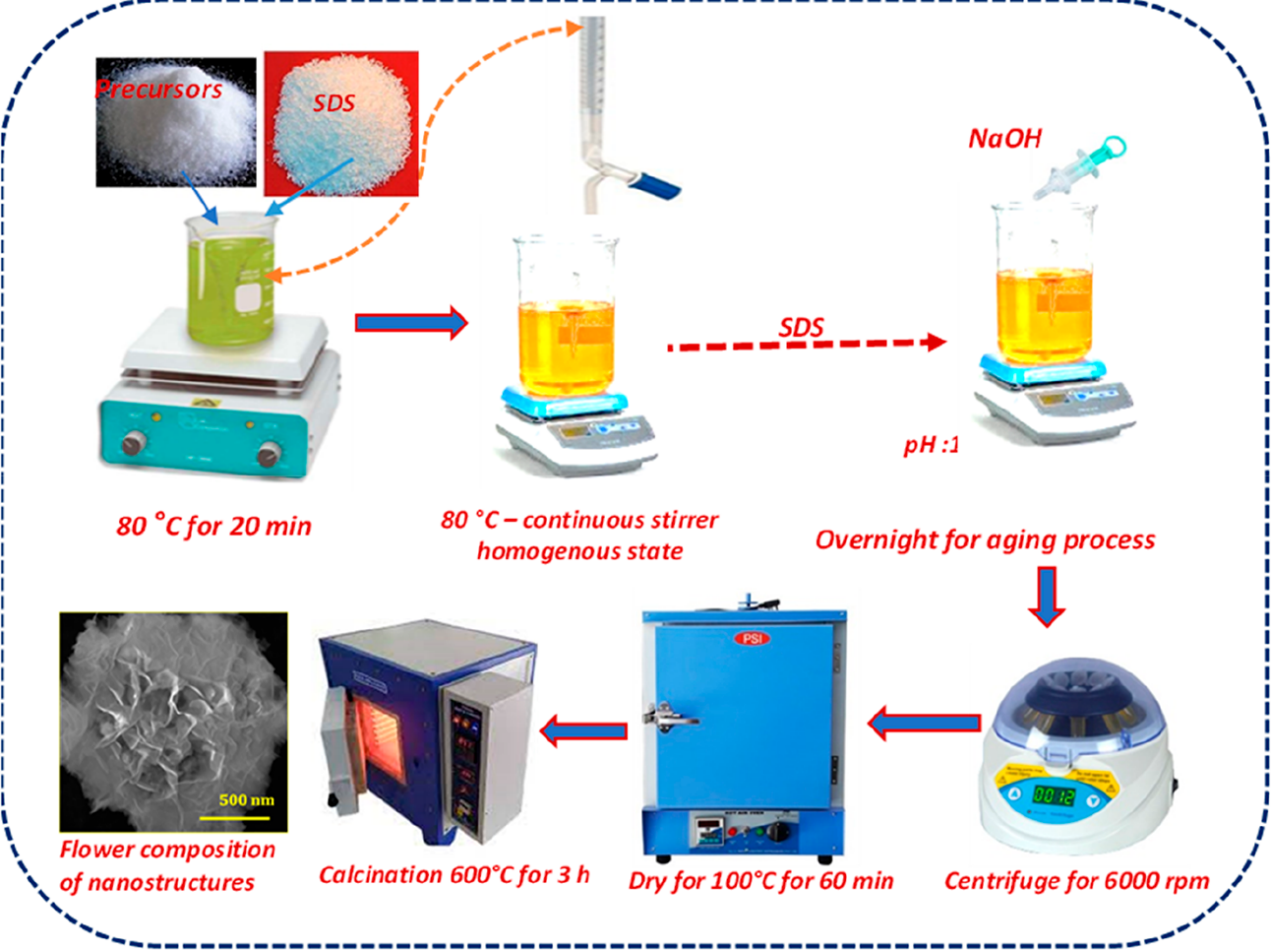
Schematic illustration of the in situ preparation of Ag/SrFeO_3_ flowers consisting of nanostructures.

In this research work, a facile hydrothermal process was used for the preparation of Ag/SrFeO_3_ nanostructures. 0.5 M of SrNO_3_, 0.25 M of FeNO_3_, 0.5 M of AgNO_3_, and 0.25 M of SDS were stirred separately in 20 mL of deionized water for an hour. All the above solutions were mixed under constant stirring. Then 1.5 g of NaOH was added into the above solution and further stirred for 30 min. The above yellow color mixture was transferred to a stainless-steel autoclave and kept in a hot-air oven at 180 °C for 18 h. After completion of the reaction, the as-prepared samples were washed with DI water/ethanol numerous times and then dried at 100 °C for 1 h in a vacuum oven to remove water contents, followed by calcination at 600 °C for 3 h, to obtain Ag/SrFeO_3_ powder and was used for further characterization. The in situ preparation mechanism of Ag/SrFeO_3_ has been schematically illustrated in Figure 1.

### 2.3 Characterization Instruments

The surface morphology and particle size of as-prepared materials were investigated by scanning electron microscopy with elemental analysis (SEM; JEOL 6500F) and high-resolution transmission electron microscopy (HRTEM; Shimadzu JEM-1200 EX). The crystal structure and phase information were examined by XRD diffraction (XRD PANalytical BV.) with Cu Kα radiation. Fourier transform infrared analysis (JASCO 6100 FTIR spectrometer) was performed within the 4000–400 cm^–1^ spectral domain, with a resolution of 4 cm^–1^, to identify interaction and functional groups. The surface area was analyzed by nitrogen adsorption–desorption in Micromeritics ASAP 2020. The prepared samples were degassed at 180 °C prior to nitrogen adsorption measurements. The isotherms were used to determine the pore size distribution by the BJH technique.

### 2.4 Fabrication of Ag/SrFeO_3_ as the Modified Working Electrode

The bare glassy carbon electrode (GCE) was continuously polished with a 0.05 μm alumina powder to a mirror-polished surface and then washed with DI water. 10 μL of Ag/SrFeO_3_ nanostructure suspension was drop-cast onto the electrode surface and dried at room temperature to form a modified GCE. Then, the modified electrode was softly rinsed with DI water to eliminate the lightly involved particles and employed for further electrochemical studies.

### 2.5 Electrochemical Studies

The electrochemical performance was confirmed and recorded using a potentiostat–galvanostat electrochemical workstation (VSP 300 Biologic Instrument). The prepared solution was purged with nitrogen gas for 10 min prior to the electrochemical experiments. The EC measurements were studied in a conventional two-compartment, three-electrode cell with a mirror-polished glassy carbon 0.07 cm^–2^ as the working electrode, Pt wire as the counter electrode, and 3 M KCl Ag/AgCl as the reference electrode.^20^ All the electrochemical measurements were carried out in PBS (pH = 7.2) under a nitrogen atmosphere at RT.

## 3 Results and Discussion

### 3.1 Surface Engineering and Particle Nature of Ag/SrFeO_3_

**Figure 2:**
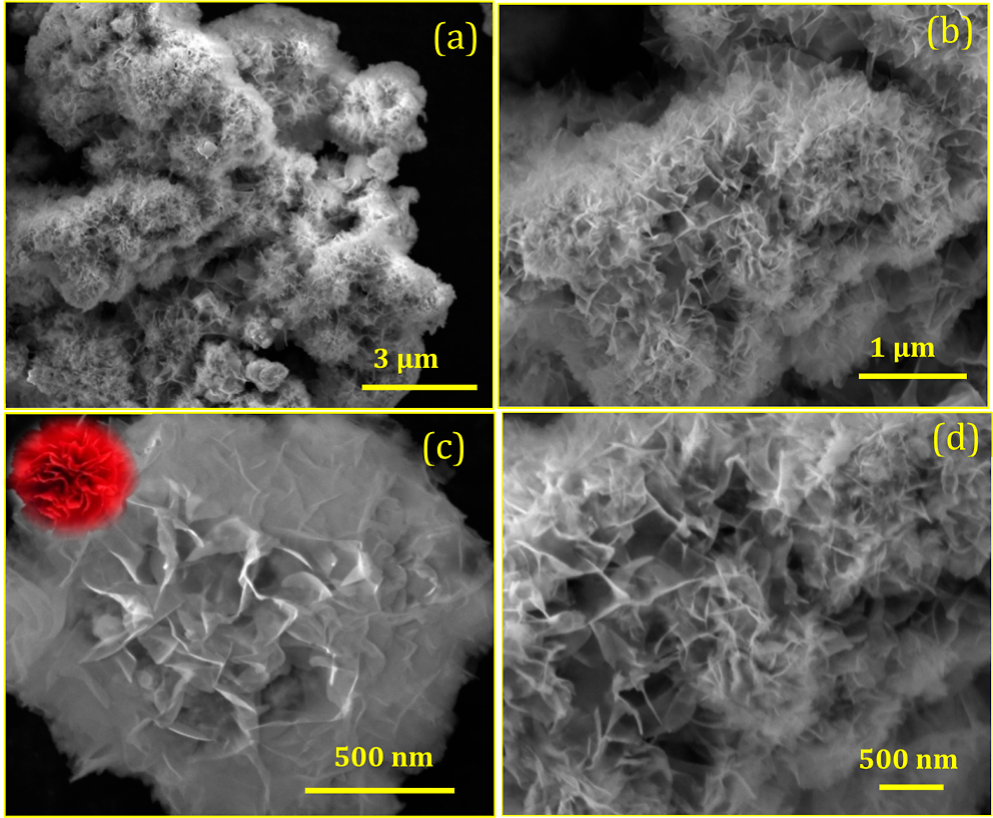
(a,b) Low-magnification image, (c) single flower-like morphology (inset: photo image of flower-like nanostructures), and (d) high-magnification surface morphology of flower-like Ag/SrFeO_3_ nanostructures.

The surface morphology and size of the particle play a crucial role in the revolution of reaction active sites for electrochemical applications. Figure 2a,b confirms the low- and high-magnification images of Ag/SrFeO_3_ flower-like morphologies with an average size of ∼2–2.5 μm. A clear formation of an individual floral pattern, as shown in Figure 2c, confirms that the flowers are formed by numerous interconnected nanosheets with an average length of ∼1 μm and a diameter of ∼80 nm. Amid the interconnected sheet, voids and interspaces can be located and the surfactant has an important role in the construction of nanosheets and assemblies from a single center, thereby acquiring the formation of the flower structures via the self-assembly hydrothermal process.^21^ This process involves two stages: nanosheets are formed rapidly in the early stage, followed by a relatively slow aggregation of the petals into flower-like structures. Moreover, the reaction temperature is a crucial factor during the synthesis process of Ag/SrFeO_3_ flower morphology. Interestingly, a close observation of Figure 2d showed that the Ag/SrFeO_3_ could offer a very short ion diffusion length in the intercalated nanosheets, which can be used as an excellent conductive pathway with large surface-to-volume ratios for electrochemical detection applications.

**Figure 3:**
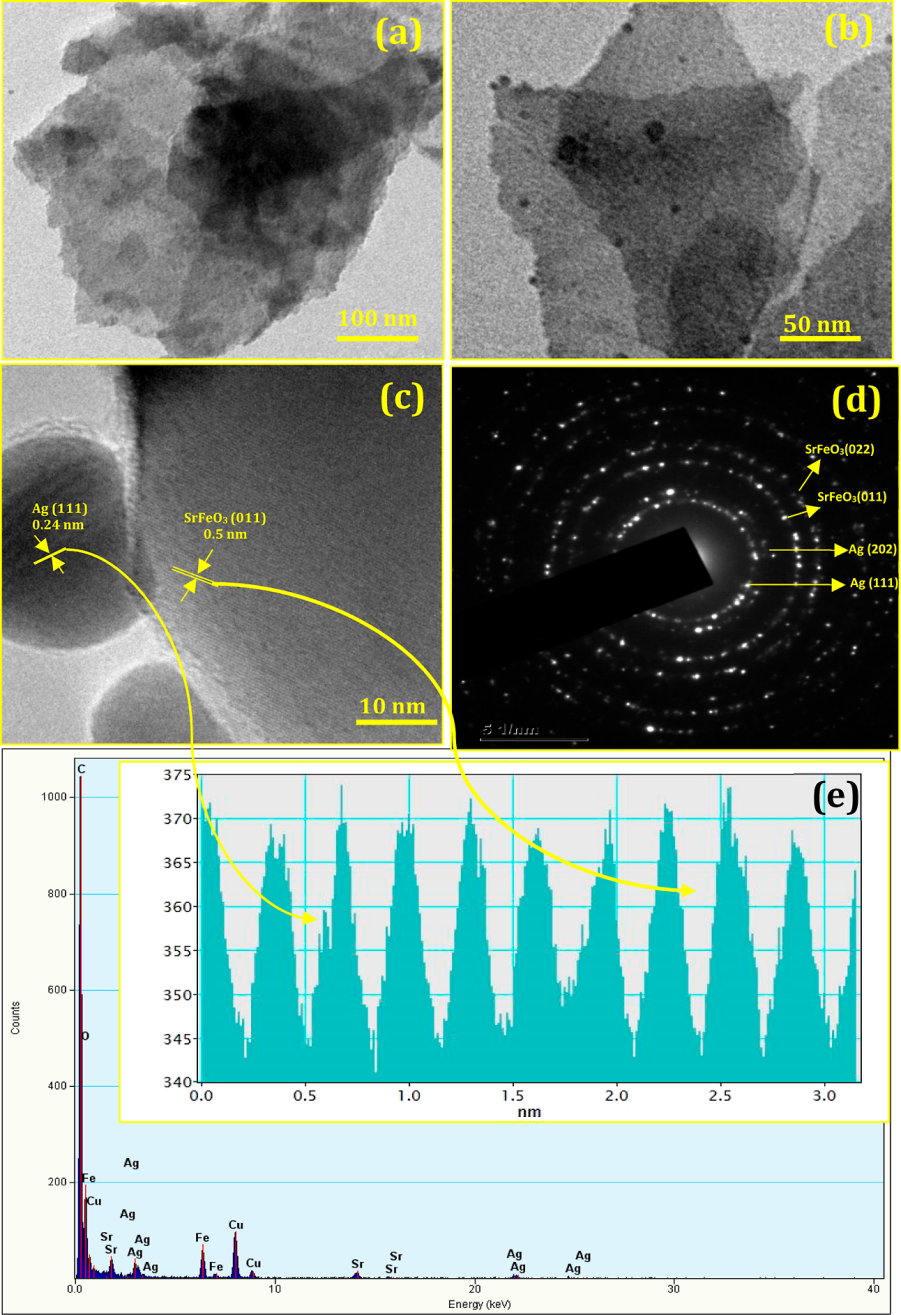
(a) Low- and (b) high-magnification of TEM images, (c) HRTEM image, (d) SAED pattern, and (e) EDAX analysis (inset top) corresponding lattice interplanar spacing of flower-like Ag/SrFeO_3_ nanostructures.

In addition, an in-depth understanding of flower-like Ag/SrFeO_3_ nanostructures was investigated through TEM, HRTEM, selection area diffraction (SAED) pattern, and EDAX analysis. A low-magnification TEM image, as shown in Figure 3a, indicates that the nanosheets aggregate to form flower-like nanostructures with an average size of ∼80 nm. The width of the nanosheets forming a floral pattern was found to be uniform along its entire length, as evidenced by the TEM image. Furthermore, in the HRTEM image (Figure 3b), nanometer-sized pores can be observed on the surface of the nanosheets. These morphologies with large surface-to-volume ratios are promising for mass and the rapid transfer of electrons during electrochemical detection. The pores might be formed during the recrystallization process or the elimination of water from constitutional OH^–^groups.^22^

The morphology was studied in detail using a high-resolution TEM image for an enlarged portion of self-assembled flower Ag/SrFeO_3_ nanostructures shown in Figure 3c. The clearly resolved lattice fringes of the (111) plane reveal that silver shows face-centered cubic (fcc) structures with a *d* spacing of 0.242 nm and the SrFeO_3_ (011) plane with orthorhombic structures with a *d* spacing of 0.5 nm (indicated between two arrowheads in Figure 3c). The diffraction rings are intermittent and consist of relatively sharp spots, as shown in Figure 3d, which indicate good crystallinity of flower-like Ag/SrFeO_3_ nanostructures as observed from the SAED pattern. The major diffraction spots correspond to Ag(111) (202) with a cubic structure and SrFeO_3_ (011) (022) with a perovskite structure. No diffraction spots were attributed to the impurity phase.^23^ The energy-dispersive elemental analysis was employed to determine the composition of Ag/SrFeO_3_ nanostructures, as shown in Figure 3e. The elements present in the samples are Ag, Sr, Fe, and O with a molar ratio of 1:1:1:3, corresponding to the stoichiometric composition of Ag/SrFeO_3_. The C peak in the spectrum can be attributed to the electric latex of the SEM sample holder. These results were consistent with the XRD patterns, which clearly established the successful preparation of the flower-like Ag/SrFeO_3_ nanostructures.

### 3.2 Crystal Structural Analysis

**Figure 4:**
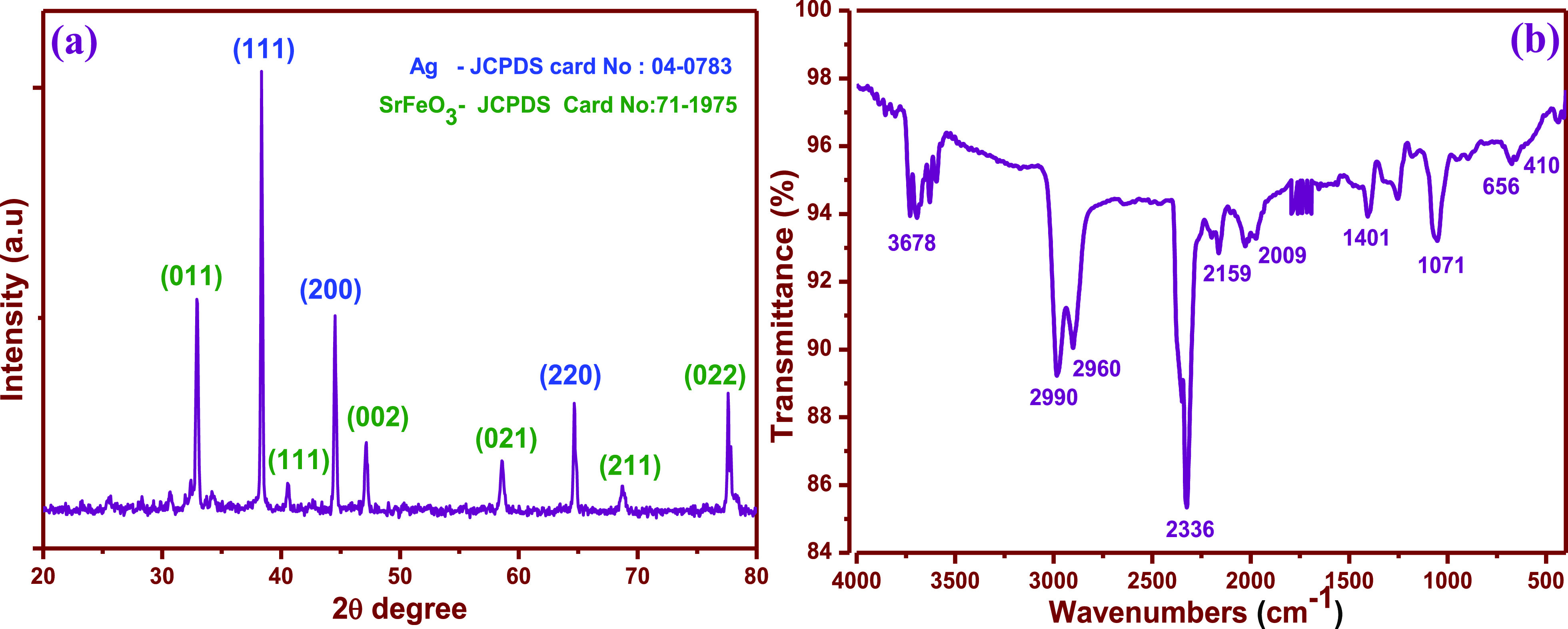
(a) XRD pattern and (b) FTIR spectra of flower-like Ag/SrFeO_3_ nanostructures.

The crystal structure and phase purity of Ag/SrFeO_3_ samples were confirmed by using an XRD pattern, as shown in Figure 4a. The XRD measurements demonstrate that all prepared samples were cubic structures (Ag) with perovskite phases (SrFeO_3_) and orthorhombic structures. The diffraction patterns are in good agreement with the JCPDS card of SrFeO_3_ (JCPDS no: 71-1975)^24^ and Ag (JCPDS no: 04-0783).^25^ No diffraction peaks from the impurities, such as the other phases of Ag and SrFeO_3_, were found within the detection limit. The strong diffraction peaks suggest that the Ag/SrFeO_3_ nanostructure samples are well crystalline, and the “*d”* spacing was 0.241 nm (Ag) and 0.50 nm (SrFeO_3_), along the crystal growth direction of the most intense peak Ag(111) and SrFeO_3_ (011), as also correlated with the results in the HRTEM analysis section. The average crystallite size of Ag/SrFeO_3_ nanostructures can be determined from Debye–Scherrer’s equation and was found to be ∼82 nm, which coincides with the TEM investigations.

The functional groups of Ag/SrFeO_3_ nanostructures were obtained from FTIR spectroscopy in an acquired range of 4000–400 cm^–1^ as shown in Figure 4b. All the observed peaks were referred from the previous literature.^26^ The spectrum band at 3678 cm^–1^ indicates the presence of the surface-adsorbed OH group or water molecules, and the band in the regions of 2990, 2960^—^, and 2336 cm^–1^ represents the symmetric vibrations that arise due to the presence of surfactants. The characteristic spectra at 2159, 2009, 1401, 1071, and 656 cm^–1^ can be ascribed to O–H bending vibration, C–C stretching bands, and CO=H stretching, respectively.^27^ The presence of the hydroxyl group plays a key role in the enhancement of sensory activities and oxygen vacancy as these functional groups perform as the main predators. The broad band at 410 cm^–1^ refers to the lattice vibration of SrFeO_3_ (Sr–O–Fe) stretching, which confirms the presence of construction of Ag/SrFeO_3_ bonding.^28^ Furthermore, Ag-oxidation and electron transfer to metal oxide were evaluated by calculating the Bader charges on the atoms before and after interface formation. Additionally, weak absorption peaks at 1750 cm^–1^ can be attributed to the absorption of moisture. These results confirm that the prepared nanostructures have no phase impurities, and the peaks corresponding to other phases were not detected, indicating the high crystal structure of the prepared samples. The result also agrees with the results discussed in the XRD section.

### 3.3 Surface Area and Pore Size Distribution Analysis

**Figure 5:**
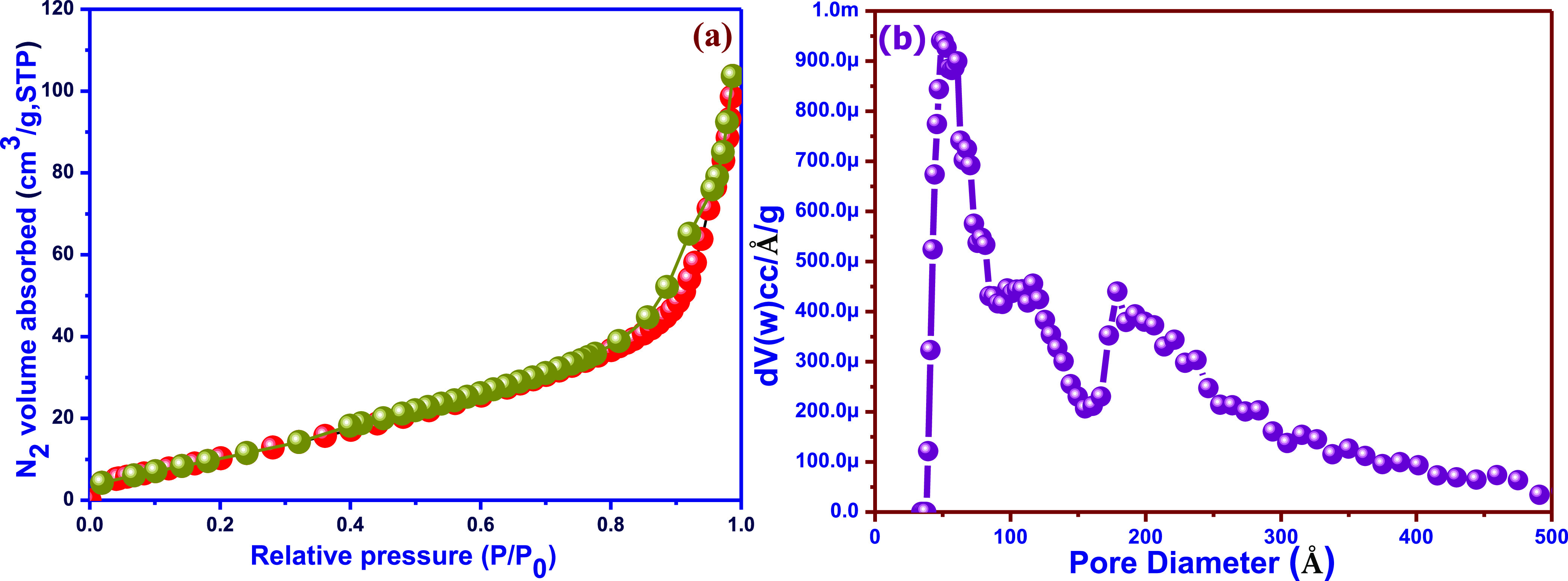
(a) N_2_ adsorption–desorption isotherms and (b) pore size distribution of flower-like Ag/SrFeO_3_ nanosheets.

The surface properties of biosensing materials are very important to achieving good sensing performance. The surface area of Ag/SrFeO_3_ nanostructures was measured using the BET and BJH methods.^29^ The nitrogen absorption–desorption isotherm BET analysis of the samples represents the type IV, H3 hysteresis loop with pressure in the range 0.8 < *P*/*P*_o_ < 1.0, which typically represents mesopores with different pore sizes as presented in Figure 5a,b. The specific surface area of the flower-like Ag/SrFeO_3_ nanostructures is 119 m^2^ g^–1^.

It should be indicated that the flower-like Ag/SrFeO_3_ nanosheets themselves do not exhibit a microporous structure. The pores can be ascribed to the interparticle space and the interbranch space. Moreover, the whole architecture is relatively large; the surface area can likely be contributed to the surface condition of the sheets. The large surface area indicates that the flower-like Ag/SrFeO_3_ nanosheets would possess a fascinating adsorbing ability to analytes in biosensing applications.

In addition, from the Barrett–Joyner–Halenda (BJH) method, the pore volume of Ag/SrFeO_3_ nanostructure samples was found to be 0.12 cm^3^ g^–1^ (Figure 5b). In the pore size distribution curve, three different peaks can be clearly noticed. The first and second peaks positioned at 80 and 100 nm may correspond to the voids between the crystallites present in agglomerated particles. The third peak positioned at 200 nm represents larger pores with a wide pore diameter distribution and can be attributed to the space between the intercrossed Ag/SrFeO_3_ petals. The presence of nanosheets in the Ag/SrFeO_3_ flower structures with varying sizes has been confirmed previously in this study from the obtained surface morphology analysis. Moreover, the whole architecture has a large surface-to-volume ratio, which can likely be contributed to the surface condition of the nanostructures, and from the Ag that has the adsorbing ability to the analyte during biosensing. These are beneficial to accomplish an enhanced electrochemical performance, which will be confirmed in the upcoming sections.

### 3.4 Electrocatalytic Activity of Flower-like Ag/SrFeO_3_ Nanostructures toward CA

#### 3.4.1 Determination of the Modified Electrode Active Area

**Figure 6:**
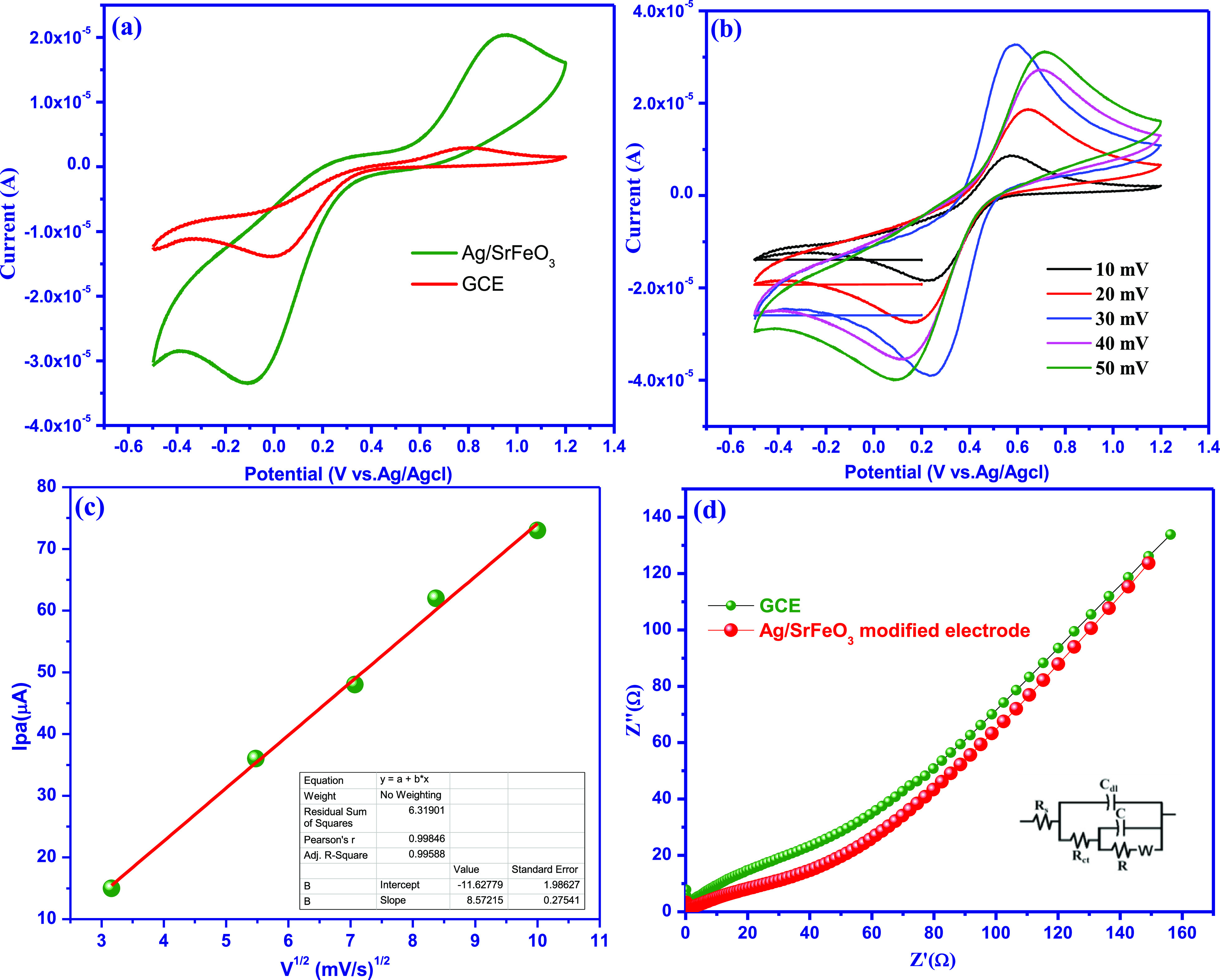
(a) CV responses of bare electrodes and modified Ag/SrFeO_3_-nanostructured electrodes. (b) Effect of various scan rates on Ag/SrFeO_3_ nanostructure electrodes. (c) shows the calibration plots of *I*_pa_ and *I*_pc_ vs υ^1/2^ and (d) EIS responses of bare Ag/SrFeO_3_ and Ag/SrFeO_3_ electrodes. All in the presence of a 2 mM K_2/3_[Fe(CN_6_)^3–4–^] complex containing 0.1 M KCl at υ = 50 mV·s^–1^.

From the science and technology point of view, it is very important to design electrochemical biosensors with high sensitivity, stability, and good efficiency toward the detection of CA in real-time applications. The typical ferricyanide/ferrocyanide redox pair was used to investigate the electrochemical properties of the bare electrode and in the modified Ag/SrFeO_3_ electrodes of a 0.1 M KCl buffer solution, as shown in Figure 6a. For the Ag/SrFeO_3_ electrodes, the redox pairings exhibited anodic peak potentials at *E*_pa_ = 0.94 and cathodic peak potentials at *E*_pc_ = – 0.10 V. The calculated peak-to-peak potential (*E*_o_′) values for bare GCE and Ag/SrFeO_3_ electrodes were 128 and 437.8 mV, respectively. The enhanced *E*_o_′ values for the modified Ag/SrFeO_3_ electrodes provide substantial information on the behavior of the substrate closer to the metallic conductive electrode in terms of electron transport. The calibration displays the anodic peak current values (*I*_pa_) for scan rates ranging from 10 to 100 mV against the square root of *V* versus *I*_pa_ values shown in Figure 6b,c. The Randles–Sevcik equation was used to determine the electrochemical active surface area (ECSA), *A* (cm^2^) in the following equation^30^
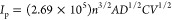


Here, *I*_p_ is the redox peak current, *n* is the number of electrons, *A* is the electrochemical surface area (cm^2^), *D* is the diffusion coefficient (cm^2^ s^–1^), *v* is the scan rate (V s^–1^), and *C* is the concentration of [Fe(CN)_6_]^3–/4–^ (mol cm^–3^). The ECSA values were calculated to be 0.026 and 0.097 cm^2^ for the bare electrode and modified Ag/SrFeO_3_ nanostructure electrodes, respectively. The results indicated that the Ag/SrFeO_3_ owns a larger active surface area, leading to a higher peak current as observed in Figure 6c.

The electrochemical impedance spectroscopy (EIS) experiment was used to find the electrical conductivity and evaluate the interface properties of the electrode in Figure 6d. The impedance data can be described concisely by the equivalent circuit model as shown in Figure 6d (inset) and the applied frequency is in the range of 1 MHz to 1 Hz with a 5 mV amplitude. The findings demonstrate that the modified Ag/SrFeO_3_ nanostructure electrode has low charge-transfer resistance (*R*_ct_) values of 12.6 Ω and good conductivity, which is much less than the *R*_ct_ value of the bare GCE (16.5 Ω). The outstanding catalytic activity of the Ag/SrFeO_3_ electrode can be explained by the rapid transfer nature of the electrode, as observed from the EIS data. Therefore, it can be ensured that the higher electrical conductivity and larger ECSA of Ag/SrFeO_3_ nanostructures can serve as a promising electrochemical sensor for CA detection.

### 3.5 Electrochemical Performance of Ag/SrFeO_3_ to CA Detection

**Figure 7:**
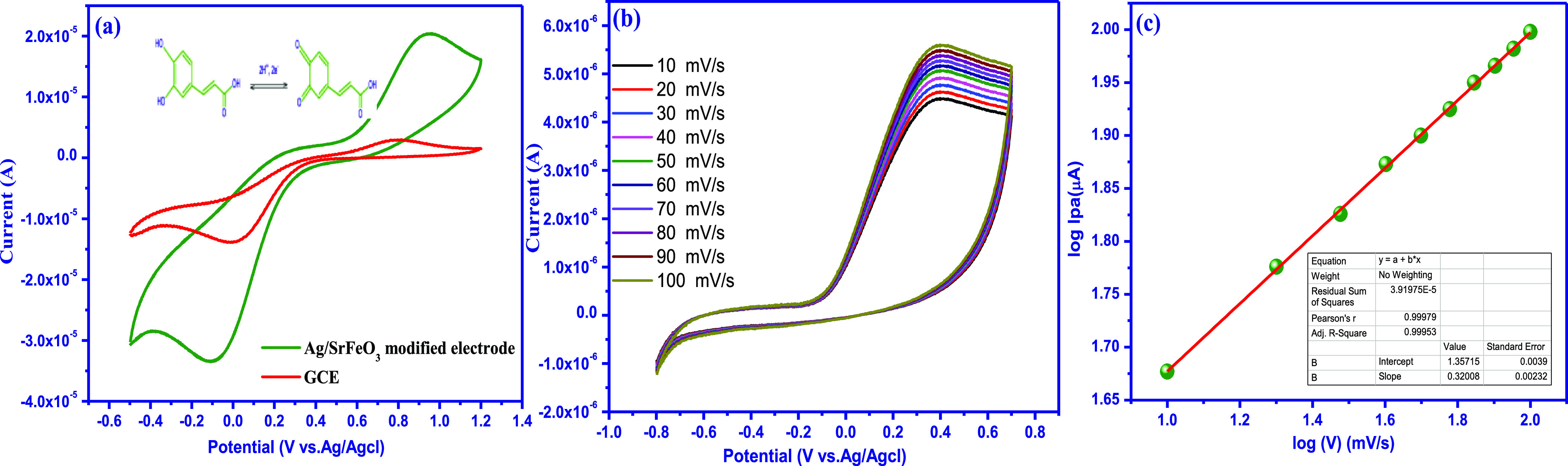
(a) CV responses of bare and modified Ag/SrFeO_3_ electrodes in the presence of 100 μM CA, in a 0.1 M PB solution (pH 7.0) at υ = 50 mV s^–1^ (inset possible mechanism of CA), (b) modified Ag/SrFeO_3_ electrodes for different scan rates from 10 to 100 mV s^–1^, and the (c) calibration plot for log υ vs log I*pa*.

The electrochemical performance of bare GCE and modified Ag/SrFeO_3_ electrodes was put through electrochemical tests utilizing CV in the potential range of −0.8 to 0.7 V vs Ag/AgCl in the pH 7.2 PB solution, with 100 μM CA. As can be observed in Figure 7a, the Ag/SrFeO_3_ electrode did not exhibit any redox activity. The Ag/SrFeO_3_ modified electrode was exposed to CV, and it displayed an oxidation peak at 0.39 V and an *I*_pa_ value of 43 μA. According to electrochemical characteristic determination investigations, the modified Ag/SrFeO_3_ electrode produced high faradaic currents with wide reduction peaks, which supports the shift in electron mobility caused by the larger grains and electrochemically active surface area.^31^ The current response of CA on the bare GCE is relatively weak, whereas the Ag/SrFeO_3_ nanostructure showed a significantly enhanced intensity. Moreover, the kinetics of the electrochemical reaction was investigated by the effect of the scan rate on the redox peak current and potential.^32,33^ The modified Ag/SrFeO_3_ electrode, which had a larger oxidation current, was exposed to CV in the presence of 100 μM CA for scan rates ranging from 10 to 100 mV/s in order to determine the electron transfer mechanism. The current values increased linearly along with the rising oxidation peak V as shown in Figure 7b. The slope value of 0.3208 on a calibration plot between log *V* and log *I*_pa_ is closer to the optimum value of 0.5 for the diffusion-controlled electron transfer behavior of CA (Figure 7c). The findings show that the *I*_pa_ for CA oxidation grew linearly and switched to a further positive potential, demonstrating a cross-exchange activity between the oxygen functional group of the modified and the Ag/SrFeO_3_ electrode and CA diffusion, which suggests an electrochemical catalytic mechanism. This linearly dependent relationship indicates that the electrochemical reaction of CA on the modified Ag/SrFeO_3_ electrode is an adsorption-controlled process. Furthermore, we confirm that the Ag/SrFeO_3_ nanostructures can be used as a potential electrochemical sensor for detecting CA in real samples.

### 3.6 Stability, Repeatability, and Reproducibility

**Figure 8:**
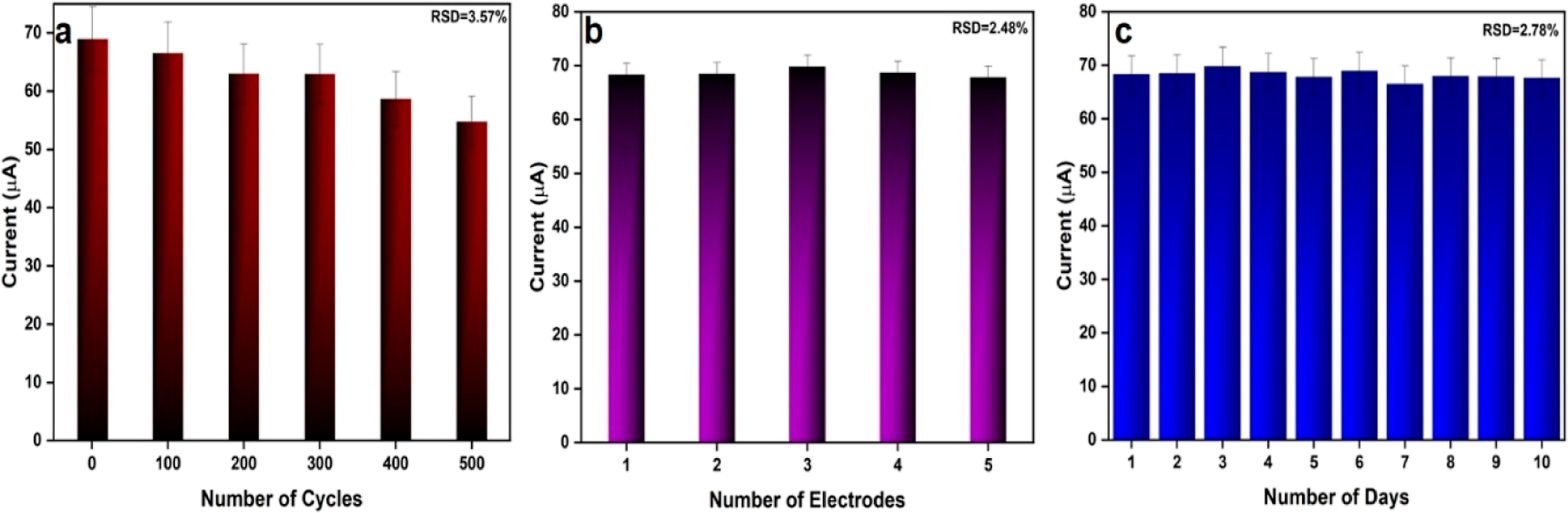
(a) Stability, (b) repeatability, and (c) reproducibility of modified Ag/SrFeO_3_ electrodes.

The stability, repeatability, and reproducibility of electrochemical sensors are particularly important in the real-time inspection of real samples. The stability of the electrode was verified by subjecting the Ag/SrFeO_3_ electrode to continuous 500 cycles in pH 7.2 PBS, where the electrode exhibited a relative standard deviation (RSD) value of 3.57%, as shown in Figure 8a. For the repeatability analysis, the Ag/SrFeO_3_-modified electrode was tested for 10 different days, which resulted in an RSD value of 2.78%, as shown in Figure 8a. In addition, for analyzing the electrode reproducibility, five different Ag/SrFeO_3_-modified electrodes were prepared under the same optimal parameters and subjected to CV under optimal conditions in 100 μM CA, which resulted in an RSD value of 2.48%, as in Figure 8c, revealing high reproducibility. Therefore, considering the repeatability, stability, and reproducibility of the Ag/SrFeO_3_ electrodes in the neutral pH and to analyze the CA oxidation behavior in physiological conditions, Ag/SrFeO_3_ electrodes are subjected to further studies.

### 3.7 Concentration Effects and Interference Studies

**Figure 9:**
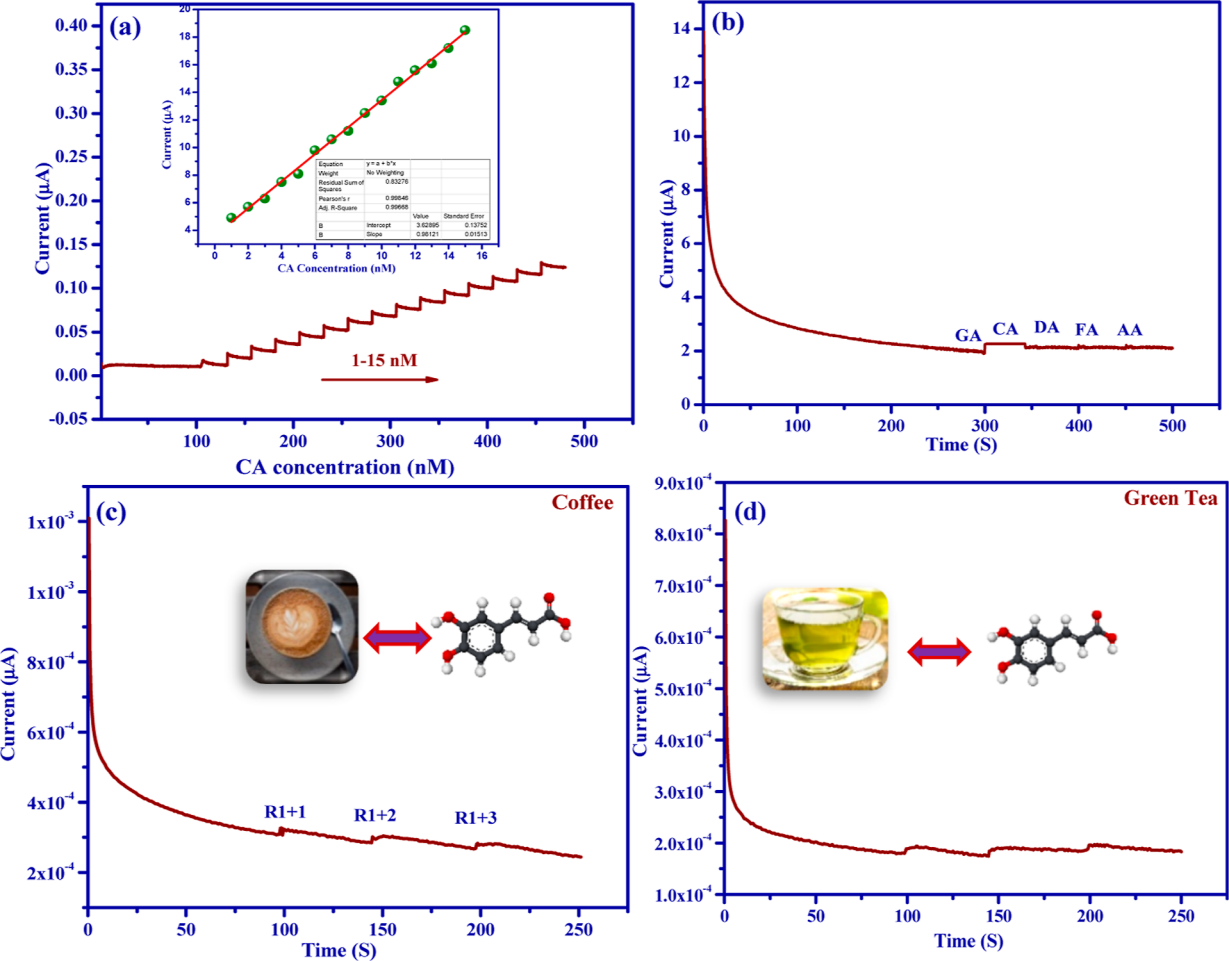
Chronoamperometric responses (a) modified flower-like Ag/SrFeO_3_ nanostructure electrode in various CA concentrations ranging from 1 to 15 nM in 0.1 M PB, and the inset illustrates the calibration plots of *i–t* peak current response and (b) selectivity measurement of potentially chemical interfering compounds at 0.39 V. All in 100 nM concentrations. Real sample measurements of CA oxidation on a flower-like Ag/SrFeO_3_ nanostructure electrode from (c) coffee and (d) green tea (inside: CA molecular structures).

The chronoamperometric technique is a promising method to inspect the electrochemical activity of modified electrodes and calculate the essential electrochemical parameters such as limit of detection (LOD), linear range, and sensitivity of the target analyte. The modified Ag/SrFeO_3_ electrodes were studied through chronoamperometric with CA concentrations ranging from 1 to 15 nM at 0.39 V; the sensitivity was 98.121*y* ± 0.0513 μA/nM, with the lowest detection limit value of ∼23 nM as shown in Figure 9a. Furthermore, the image inset in Figure 9a displays the calibration curve between CA concentration and its current value. According to the literature for other analytes,^33−35^ our findings showed that the produced flower-like Ag/SrFeO_3_ nanostructures are highly appropriate for electrochemical sensing applications of CA. The selectivity of electrochemical sensors is most significant in the presence of various possible interfering compounds. Thus, numerous possible interfering substances were investigated. The selectivity of the developed Ag/SrFeO_3_ electrode was evaluated with possibly interfering chemical compounds of CA, GA, FA, AA, and DA in order to assess the practical applicability of the electrode. It is remarkable that the electrode demonstrated extreme selective detection of all the investigated chemicals in the selectivity test utilizing amperometry at analyte concentrations of 150 nM, as shown in Figure 9b. This revealed that the present Ag/SrFeO_3_ electrode sensor has excellent selectivity for CA detection.

### 3.8 Real Sample Analysis

In this experiment, two different real samples without any pre-treatment were measured by CV as shown in Figure 9c,d; furthermore, the results are summarized in Table 1. The Ag/SrFeO_3_ electrode was subjected to CA in the coffee (R1 + 1, R1 + 2, and R1 + 3) and green tea (R2 + 1, R2 + 2, and R2 + 3) samples using the CA method in order to assess the real sample application usage of the electrode.

**Table 1:** Determination of CA Levels in Coffee and Green Tea Samples on an Ag/SrFeO_3_ Electrode

real sample	spiked (nM)	detected (nM)	recovery (%)
coffee (R1)	50	48.4	96.8
	100	98.5	98.5
	150	149.7	99.8
green tea (R2)	50	46.2	92.4
	100	97.5	97.5
	150	147.8	98.53

Under conventional addition procedures and 200 rpm hydrodynamic conditions, the CA concentration was changed from 50 to 150 nM. Table 1 provides a summary of the findings, which are confirmed in Figure 9c,d. All CV measurements were carried out three times to give an average value of CA concentration. The concentration of CA in coffee was determined to be 48.4–149.7 μM, and in tea, it was found to be 46.2 to 147.8 μM. Furthermore, upon the addition of a certain amount of CA, the recovery rate of CA was found to be in the range of that of the flower-like Ag/SrFeO_3_ nanostructure electrode showing notable spurious recovery values between 96.8 and 99.8% and 92.4 and 98.53%. Meanwhile, CA could be detected in real samples, and the recommended electrode was very stable and selective. From this described considerable electrochemical response, a Ag/SrFeO_3_ nanostructure electrode was found to be an efficient electrode material for real-time applications.

The electroanalytical results of the modified Ag/SrFeO_3_ nanostructure sensor for the determination of CA are compared with those in the previous literature, as presented in Table 2. It clearly demonstrates the exclusive improvement and enhanced electrocatalytic activity of the reported Ag/SrFeO_3_ nanostructure electrode sensor toward the determination of CA, and excellent anti-interference capability, higher stability, and reproducibility are recorded. Finally, the proposed Ag/SrFeO_3_ sensor has been successfully investigated for the detection of CA in real coffee and green tea samples.

**Table 2:** Comparison of the Various Electrodes for Electrochemical Detection of CA

electrode	technique	LOD (mm)	linear range (μM)	refs
AuNPs—chitosan	DPV	25	2–350	(36)
Nafion/Tyre/carbon	amperometric	46	0.08–2	(37)
activated GCE	DPV	68	0.05–0.10	(38)
MIS	DPV	150	0.500–60	(39)
MWCNT-CS/Au	amperometric	150	0.7–10	(40)
AuNPs/GRNS	DPV	50	0.5–50	(41)
Au/PdNPs-GRF	DPV	60	0.03–350	(36)
Ag/SrFeO_3_	Chronoamperometric	23	0.001–0.010	this work

## 4 Conclusions

In summary, a new active electrode material based on flower-like Ag/SrFeO_3_ nanostructures was successfully prepared by a facile route and applied for the detection of CA in real samples. The prepared materials were scientifically characterized for structure, microscopy, and spectroscopy analysis. Remarkably, fabricated Ag/SrFeO_3_ nanostructures provide a high specific surface area, uniform shape, and particle size, which are helpful for enhanced biosensing performance. The modified Ag/SrFeO_3_ nanostructure electrode exhibits a well-defined oxidation peak with a wide range of 10–100 μm, excellent sensitivity with a detection limit of 23 nM, as well as good stability and reproducibility. Furthermore, the present sensor was successfully applied to detect CA in real samples of coffee and green tea with spurious recovery values between 99.8 and 98.53% for 150 nM. The outstanding sensing behavior could be directly accredited to the SrFeO_3_ interlayer, effectively restraining the enrichment effect of Ag nanoparticles, which possess not only a high specific surface area but also good conductivity and synergistic effects. These demonstrate that the Ag/SrFeO_3_ electrode is a promising candidate for the real-time determination of CA concentration in coffee and green tea.
